# Long chain sphingomyelin depletes cholesterol from the cytoplasmic leaflet in asymmetric lipid membranes

**DOI:** 10.1039/d1ra01464a

**Published:** 2021-06-28

**Authors:** Maria Lyngby Karlsen, Dennis S. Bruhn, Weria Pezeshkian, Himanshu Khandelia

**Affiliations:** PHYLIFE: Physical Life Science, Department of Physics, Chemistry and Pharmacy, University of Southern Denmark Campusvej 55 Odense 5230 M Denmark hkhandel@sdu.dk

## Abstract

The transbilayer distribution of cholesterol (CHL) in complex asymmetric lipid membranes remains controversial, with contrasting investigations suggesting that there is more CHL either in the exoplasmic, outer leaflet (OL) or the cytoplasmic, inner leaflet (IL) depending on cell type or model, membrane composition, and method of investigation. Here, we launch systematic coarse-grained molecular dynamics simulations to investigate the impact of the sphingomyelin (SM) acyl chain length upon CHL distribution in asymmetric lipid membrane mixtures which account for the variation of the most abundant headgroups and acyl chain unsaturation in the two membrane leaflets. We find that there is always more CHL in the OL, but longer chain SM depletes more CHL from the IL than short chain SM in simple membrane mixtures containing SM and 16 : 0, 18 : 1 phospholipids. The difference between longer and shorter chain SM is neutralised in a more complex asymmetric membrane, where there are more saturated tails in the outer leaflet. We propose that interdigitation of long-chain SM into the opposing IL pushes cytoplasmic CHL towards the OL, but higher chain saturation of the outer leaflet compensates for the effect of SM chain length.

## Abstract 2

Long acyl chain sphingomyelin and saturated phospholipid tails in the outer membrane leaflet deplete cholesterol from the inner leaflet in mammalian membranes.

## Graphical abstract

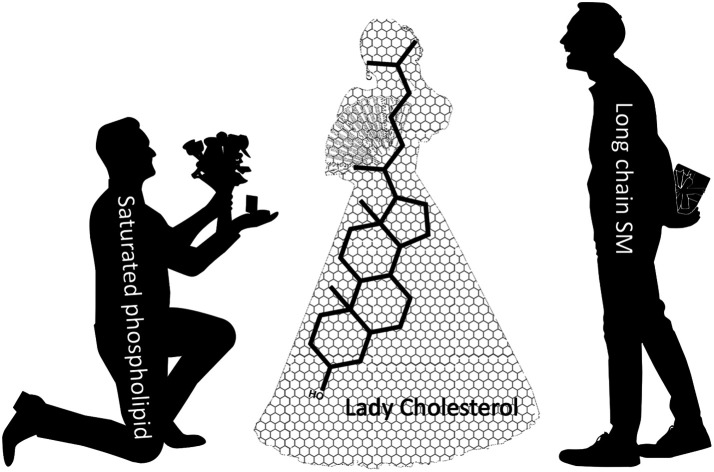

## 1 Introduction

CHL is an important and the most abundant (30–50% of all lipids) molecule in the plasma membrane (PM) that surrounds all eukaryotic cells. It has several vital roles in the PM *e.g.* maintaining membrane integrity, modulating membrane fluidity over the range of physiological temperatures, forming PM nanodomains which putatively serve as hotspots of biochemical reactions and binding events,^1^ and influencing the conformation and function of several key classes of membrane proteins including G-protein coupled receptors, mechanosensitive ion channels and ion pumps.^2–6^ While the concentration of the CHL in the cell and different cellular membranes has been well studied, its trans-bilayer distribution remains controversial.^7^ An even distribution of CHL molecules in the two leaflets is expected in a flat, symmetric bilayer. However, the PM of eukaryotic cells is highly asymmetric with respect to the lipid composition of the two leaflets (trans-bilayer asymmetry). The asymmetry drives important cellular functions and pathways. Most of the sphingomyelin (SM) and phosphatidycholine (PC) lipids are within the outer, exoplasmic leaflet (OL) while the inner, cytoplasmic leaflet (IL) mostly consists of the phosphatidylethanolamines (PE), phosphatidylserine (PS) and phosphatidylinositol (PI) lipids.^7,8^ Trans-bilayer asymmetry is not an equilibrium configuration of a membrane (at equilibrium, an even distribution is expected). The cell uses active processes carry out by flippase proteins to maintain trans-bilayer asymmetry because there is a large free energy barrier associated with the translocation of a charged lipid from one bilayer leaflet to the other across the membrane hydrophobic core. The transbilayer distribution of the major phospholipid components of the PM can now be carefully determined using highly sensitive lipidomics experiments.^9^ However, data pertaining to the distribution of CHL across the two bilayer leaflets remains contradictory owing to the low free energy barrier of CHL to flip flop across the two leaflets. Some results show that most of the CHL molecules are concentrated in the IL while others suggest that the majority exist within the OL, reviewed in ref. 10 and 11. It has also been suggested that CHL distribution depends on the cell type and age.^12^ The distribution of CHL across the bilayer leaflets is likely to be determined by a passive thermodynamic equilibrium distribution that equalizes the CHL chemical potential in the two leaflets. An active process that maintains CHL asymmetry (if it exists) is unlikely considering the rapid flip–flop of the CHL, and remains to be discovered. Based on the above arguments, a theoretical model suggested that most CHL molecules accumulate in the IL due to the negative curvature of the PE lipids present in this leaflet.^10,11^ Another mechanism that can also drive CHL to the IL is the presence of the PS and PI lipids, because a coarse grained (CG) simulation study demonstrated that CHL has a higher affinity to the charged leaflets.^13^ However, the same simulation study suggested that CHL prefers saturated lipids, which are more abundant in the OL. Another factor that can drive CHL to the OL is the presence of SM lipids that exclusively exist in this leaflet. Theoretical models predict that 80% of CHL resides in the OL owing to the presence of SM, but contributions from the bending energy reduce this abundance to 63%.^14^ The majority of SM in mammalian cells in the outer leaflet have a long C24 acyl chain,^9^ which can interdigitate into the opposing leaflet.^15,16^ However, there are also more than 4% short-chain SM in the outer leaflet. In the inner leaflet, the small fraction (less than 0.1%) of SM are all exclusively short-chain lipids.^9^ Mutations which replace C24 SM by C18 SM have physiological consequences related to obesity and insulin resistance.^17,18^ We previously showed that a delicate balance between SM–CHL hydrogen bonding and interactions with the solvent result in weaker interactions with C24 SM with CHL, compared to shorter chain C18 SM lipids.^16^ At the same time, free energy calculations showed that CHL preferred the OL in the presence of C18 SM and the IL in the presence of C24 SM.^16^ However, no PE or PS lipids were present in the lower leaflet in these all-atom simulations. In addition, all-atom simulations suffer from the constraint that CHL cannot spontaneously flip flop on the simulated timescales (100 s of nanoseconds).^19^

Overall, one can argue that SM and saturated lipids pull more CHL into the OL, while PE and charged lipids attract more lipids to the IL. The competition between these determinants decides the equilibrium distribution of the CHL in the PM. Here, we exploit the possibility of spontaneous CHL flip–flop in molecular dynamics (MD) simulations employing the CG MARTINI force field for lipids, to investigate the effect of SM chain length on the distribution of CHL in the two bilayer leaflets with compositions reminiscent of biological membranes, but simplified to eliminate minor lipid species. The flip–flop rate of CHL in simulations of CG lipid bilayers can range from 1–20 μs^−1^,^20–23^ depending on the presence of unsaturated lipids (more unsaturated lipids increase flip–flop rates), cholesterol content (more cholesterol lowers flip–flop rates) and overall bilayer composition (overall, a more ordered bilayer will reduce CHL diffusion and flip–flop rates). Simulations of asymmetric membranes have to be set up with care to ensure either that the tension in each leaflet is zero, or equivalently, the area of each leaflet is identical.^24^ When investigating CHL asymmetry, if the overall area of each leaflet is not calculated carefully, CHL will simply accumulate into the leaflet containing too few lipids. We therefore employ a careful methodology which minimizes this area difference (described in detail in the Methods section). We find that long-chain SM lipids interdigitate into the IL, and push more CHL into the OL to achieve mechanical equilibrium and balance the free volume in the two leaflets.

## 2 Methods

### 2.1 Composition of asymmetric bilayers

We chose our bilayer compositions based on the lipidome of human erythrocytes,^9^ simplified to eliminate minor lipid species and all proteins. Two compositions were chosen. First, a simple asymmetric membrane was modelled with predominantly PS and PE in the IL, and PC and SM predominantly in the OL. The lipid acyl chains were set to 16 : 0–18 : 1 for PS, PE and PC, while the SM was either all 18 : 1–18 : 0 (DPSM) or 18 : 1–24 : 0 (DSSM). The overall CHL fraction was 0.29, similar to prior simulations of plasma membrane models.^25^ The final system compositions are shown in Table 1. To account for complexities in acyl chain length, a complex asymmetric bilayer was also setup with the composition shown in Table 1. Lipids present at less than 5% were not included.

**Table 1:** Composition of the bilayers for the simple and complex systems. For each composition, two types of systems were simulated. One with short chain SM (18 : 1, 18 : 0), also called DPSM and one with long chain SM (18 : 1, 24 : 0), also called DSSM

Lipid Tails	Head group	% in OL	% in IL
**Simple systems**			
16 : 0, 18 : 1	PC	31.9	16.97
18 : 1, 18 : 0/24 : 0	SM	39.53	0
16 : 0, 18 : 1	PS	0	24.40
16 : 0, 18 : 1	PE	0	30.06
—	CHL	28.57	28.57

**Complex systems**			
16 : 0, 20 : 4	PC	7.25	0
16 : 0, 18 : 2	PC	16.58	10.85
16 : 0, 18 : 1	PC	6.28	5.84
18 : 1, 18 : 0/24 : 0	SM	41.32	0
16 : 0, 20 : 4	PS	0	27.62
16 : 0, 22 : 6	PE	0	9.93
18 : 1, 20 : 4	PE	0	17.20
—	CHL	28.57	28.57

### 2.2 Construction of asymmetric bilayers

Unlike symmetric bilayers, the construction of asymmetric MD models of bilayers is challenging because the total area of the two leaflets must be as close as possible to each other to avoid artifacts arising from imperfect lipid packing. For example, if one leaflet has too few lipids, cholesterol will accumulate in this leaflet to fill up the free volume and establish mechanical equilibrium. To avoid such artifacts, we devised the following workflow to eliminate area mismatch as much as possible.

To calculate optimal area per lipid (APL) of each component, we simulated symmetric bilayers, where each monolayer had the desired composition of a single leaflet of the target asymmetric bilayer. For example, for the simple systems, we ran simulations of symmetric POPC–SM–CHL bilayers of composition of the OL to obtain the most likely area per lipids of POPC, SM and CHL, and a symmetric POPC–POPE–POPS–CHL bilayer of composition of the IL, to obtain the most likely APL of POPC, POPE and POPS in the IL. The simulation box size was set to 14 × 14 × 10 nm for these simulations, and APLs were obtained from Voronoi triangulation using apl VORO.^26^ From these APLs, the number of lipids required to model a bilayer of a target total area was obtained.

We then constructed an asymmetric bilayer using the number of lipids calculated in the previous step. Such an asymmetric bilayer should have minimal area mismatch between the two leaflets, presuming that the CHL content is identical in the two leaflets. The preceding assumption is certainly not correct if CHL distribution is asymmetric, and we account for this in the following manner.

After a 10 μs production run, a trans-bilayer CHL asymmetry is created by overall flip–flop of CHL molecules to equalize the chemical potential of CHL in the two leaflets. However, such an asymmetry agains creates an area mismatch of Δ*A*_chol_ in the two leaflets, because CHL occupies additional area in the leaflet with more CHL.

To eliminate area mismatch again, we fill the CHL-depleted leaflet with additional lipids which occupy an area corresponding to Δ*A*_chol_, and repeat another 10 μs production iteration. The overall CHL concentration is kept constant across all iterations. After several such iterations, it is expected that the CHL distribution will stabilise across the two leaflets.

A second approach to minimising the area mismatch is to choose different initial ratio of CHL in the two leaflets in the starting configuration of the simulations. We launched simulations where the ratio between the number of CHL in the OL to the total number of CHL in the IL varied from 0.5 to 0.62, *i.e.* CHL was always richer in the OL, as indicated by the first set of simulations (see Results section). Note that the number of phospholipids for each leaflet remained the same. For each setup, we simulated 5 replicas with different initial velocity distributions. Only the systems with simple lipid compositions were simulated in this way. This set of simulation is referred to as the REPLICA simulations hereafter.

Overall, we have ran close to 770 μs of simulations in MARTINI time, corresponding to a real sampling time of close to 3.1 ms.^27^

### 2.3 Simulation details

The coarse-grained (CG) MARTINI force field^27–29^ is used to model the lipid bilayers (required files were obtained from the MARTINI web site).^30^ In MARTINI, 4 heavy atoms are typically represented by a single particle. For example, CHL is modelled by 8 and POPC by 12 beads. A CG force field is necessary to permit CHL flip flop to measure trans-bilayer CHL asymmetry. All lipid bilayers are modeled using the insane.py script obtained from the MARTINI web site,^30^ in combination with home-made Matlab scripts. The bilayers are solvated with non-polarizable water beads along with antifreeze beads.^27^ Na^+^ and Cl^−^ ions are present to ensure a salt concentration of 150 mM. All simulations are performed using GROMACS 2019.x.^31,32^ The system was equilibrated using the Berendsen coupling^33^ for both temperature and pressure. Production runs are carried out in the NPT ensemble for 2 μs using a time step of 20 fs. The temperature is controlled using the Nosé–Hoover thermostat,^34,35^ with a reference value of 310 K, and the pressure is coupled semi-isotropically using the Parrinello–Rahman barostat,^36^ with a reference value of 1 bar. The van der Waal's forces are shifted to zero from 0.9 nm to 1.2 nm. Electrostatic interactions are calculated using the reaction-field method as recommended.^37^

The CHL distribution is measured from the *z*-coordinate (bilayer normal) of the hydrophilic ROH bead of CHL. Note that CHL can occupy the bilayer center in MARTINI simulations, but for the sake of simplicity, all CHL molecules are assigned either to one leaflet or the other based on the position of the ROH bead. No CHL molecules are classified as being in the center of the bilayer. Lipid tail order parameters are calculated from a modified version of scripts available on the MARTINI web pages.^30^ All other quantities are measured using GROMACS tools. All reported properties are calculated for the last μs of simulation.

## 3 Results and discussion

### 3.1 CHL distribution

**Fig. 1:**
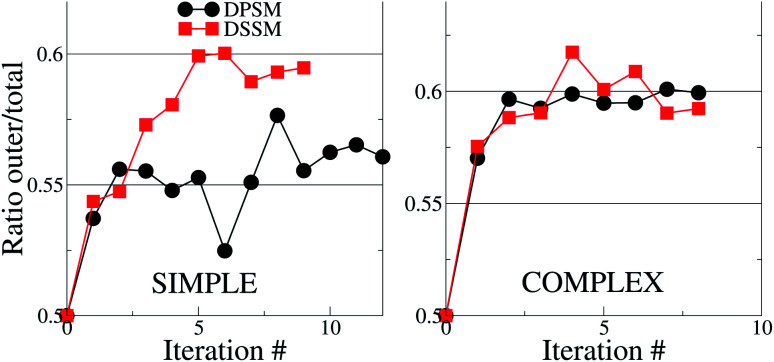
Cholesterol distribution in the simple and complex systems, compositions are listed in Table 1. The *y* – axes show the ratio between the number of CHL molecules in the OL to the total number of CHL molecules in the system. If the ratio is 0.5, then CHL is evenly distributed in the two leaflets. In both cases, there is more CHL in the OL, than in the IL. The error bars are smaller than the size of the symbols and are therefore invisible.

For the simple systems, the presence of SM in the OL attracts a higher fraction of CHL in this leaflet compared to the IL (Fig. 1). The distribution ratio of CHL in the OL *versus* the IL converges after 9 and 12 iterations of 10 μs each for the simple DSSM and DPSM systems respectively. More CHL partitions into the OL in the presence of longer chain SM (C24), than when C18 SM is present instead (Fig. 1). Since CHL flip flops rapidly in MARTINI simulations, and because CHL molecules close to the bilayer center are assigned to either leaflet, there are some uncertainties associated with the data shown in (Fig. 1). However, it is clear that first, more CHL partitions into the OL, and secondly, that SM chain length has a significant impact on the CHL distribution ratio. The flip–flop rates in our simulations are 1.95 and 3.65 per μs for the DPSM and DSSM simple simulations respectively. These numbers are averaged over all CHL molecules and correspond to a large number of flip–flop events. The higher rate in the DSSM simulation is most likely the result of more CHL flip–flopping into the outer leaflet. The numbers compare favourably to previous estimates (5.48 per μs) for systems without SM lipids.^20,38^ With SM, the flip–flop rate is expected to be slower owing to further ordering of the membrane.

For the complex systems, the distribution ratio stabilises after 9 iterations, but the trend is different from the simple systems (Fig. 1). For DSSM, the distribution ratio is similar for the simple and complex systems. For DPSM, however, more CHL distributes in the OL in the complex system. One factor which leads to more CHL in the OL is that there are less saturated lipid acyl chains in the IL (∼42% of phospholipid tails) in the simple systems, compared to the complex systems (∼75% of phospholipid tails), and CHL is known to prefer saturated tails over unsaturated tails.^13^ Transbilayer asymmetry in the distribution of saturated tails led to a significantly increased proportion of CHL in the leaflet containing saturated chains in prior CG simulations.^21,39^ The increased saturation of the OL lipids scrambles the difference between DPSM and DSSM in the complex systems.

**Fig. 2:**
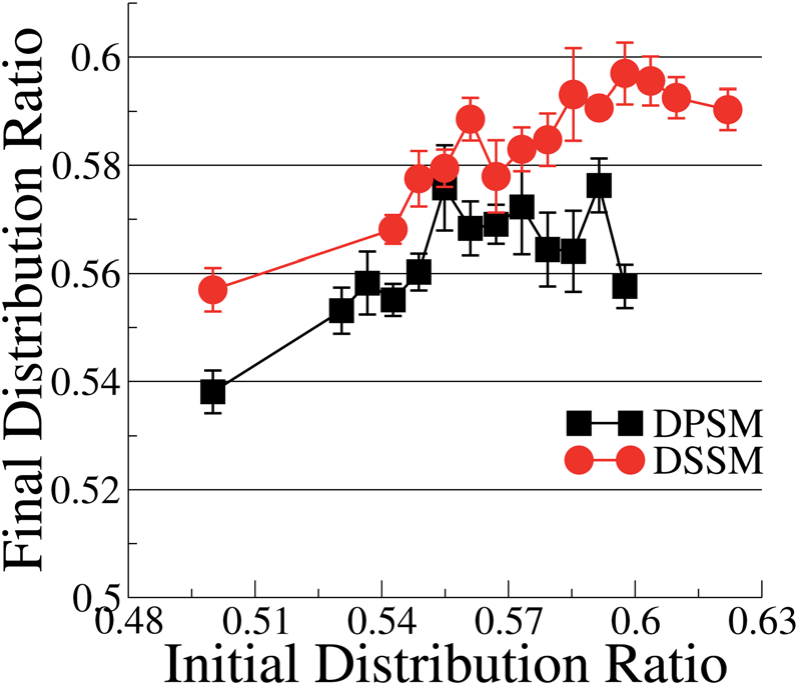
Cholesterol distribution in the systems where the OL : IL CHL ratio is initially varied between 0.5 and 0.62. The phospholipid compositions corresponds to the simple systems. The error bars are standard errors over 5 replicas.

With the exception of two points where the difference is not statistically relevant, the differences between DPSM and DSSM are also retained for the REPLICA systems where different initial CHL ratio were enforced in the starting simulated configurations (Fig. 2). In principle, different initial CHL ratio should lead to the same final CHL ratio after sufficiently long sampling. However, different initial percentages of CHL in the two leaflets lead can cause different initial areas of the two leaflets and lead to different final CHL ratio. It is therefore paramount that the simulations are carried out carefully while minimising the initial area mismatch as much as possible, as is done in the simulations with several consecutive iterations (data in Fig. 1) when the area of the OL and IL are kept equal at each instance. Furthermore, since there is always more SM than CHL in the OL, it is possible that a higher number of initial CHL in the OL leads to the formation of more stoichiometric SM–CHL complexes, thus retaining more CHL in the OL towards the end of the simulation. It is worth reiterating that the apparent lack of convergence of the data in Fig. 2 is not a consequence of limited sampling, but of problems arising out of initial area mismatch in the two leaflets.

Transbilayer asymmetry in acyl chain saturation is identical in all our simple systems. The presence of SM in the OL, and the length of the SM chain are the other two key driving forces which determine the difference in the CHL distribution ratio. In the remainder of this article, we will analyse the simple systems further in an attempt to converge on a hypothesis of why long-chain SM drives more CHL into the OL, in apparent contrast to our earlier findings using all-atom simulations in conjunction with a host of analytical techniques.^16^ For this, we first investigate if the structural properties of the bilayer are different in the DPSM (C18) and the DSSM (C24) simple membrane simulations.

For analysing bilayer properties, we chose the final iterations of the DPSM and DSSM simple system simulations. For time-averaged properties, the last 1 μs of the simulations were analysed.

### 3.2 Bilayer properties

**Fig. 3:**
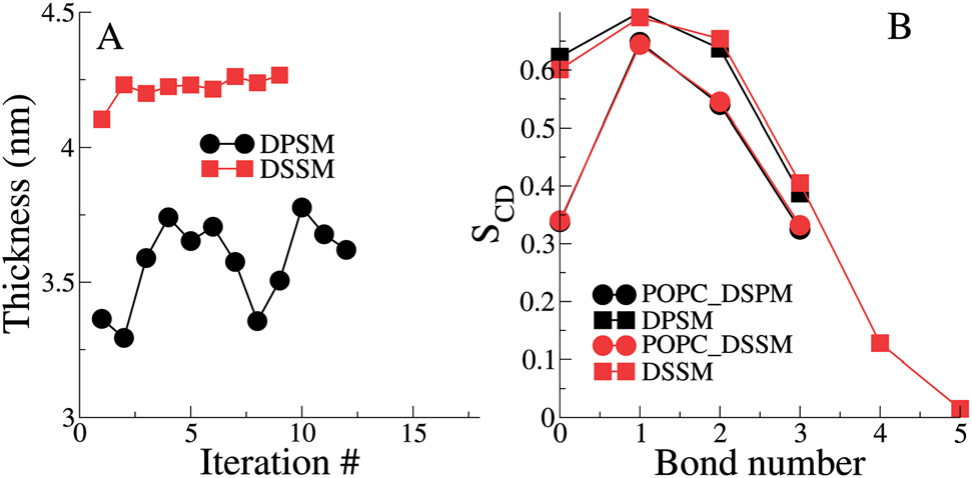
(A) The bilayer thickness for all iterations for the simple systems. Thickness is calculated as the average distance between the phosphate beads in the two leaflets. (B) Lipid tail order parameters for one of the simple DPSM and DSSM systems. For both A and B, the error bars are very small and hidden underneath the symbols.

**Fig. 4:**
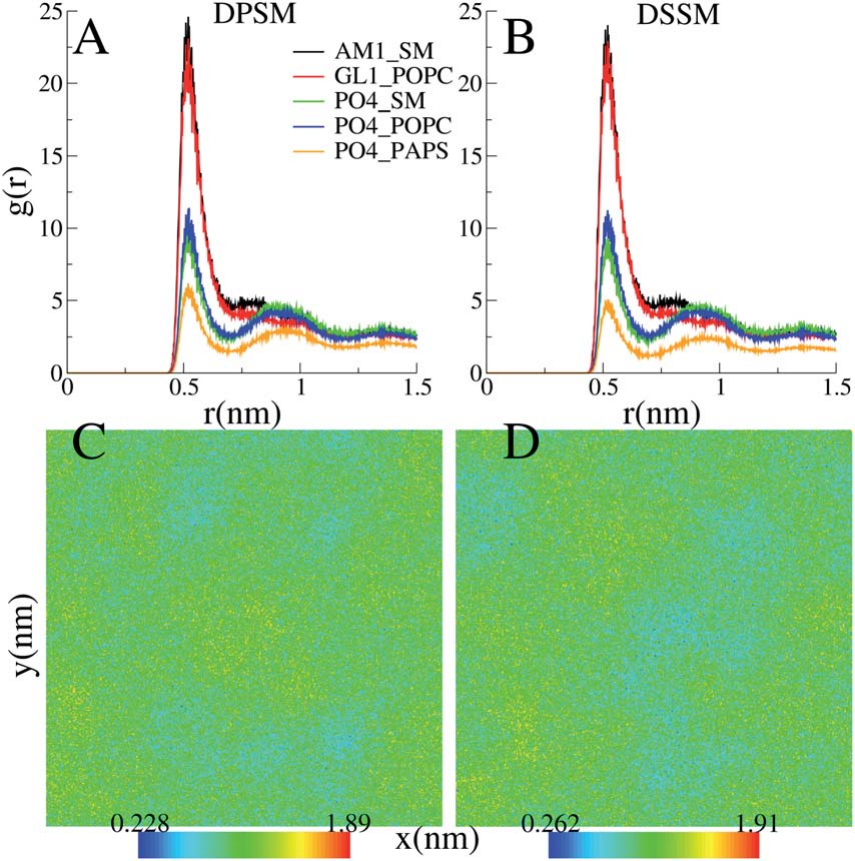
(A) and (B) Radial distribution functions between the phospholipid headgroups and the CHL headgroup bead ROH for representative DPSM and DSSM simple systems. (C) and (D) 2-D number density profiles in the *xy*-plane of SM + CHL for the DPSM and DSSM systems respectively. The grid in parts (C) and (D) is 12 nm × 12 nm.

The thickness of the bilayers is calculated from the average distances between the phosphate beads in the two leaflets. The thickness of the longer chain DSSM containing bilayers is expectedly higher, by about 0.5 nm (Fig. 3). There is an increase in the thickness over the first few iterations, probably resulting from more CHL moving the OL, and forming nanodomains with SM lipids (see Fig. 4 later), straightening them in the process, resulting in increased membrane thickness.

The lipid tail order parameters demonstrate expected trends (Fig. 3B). The SM tails have higher order parameters compared to the POPC lipid tails. Slightly higher order parameters in the DSSM containing bilayers near the bilayer center result from the interdigitation of the DSSM acyl tails into the opposing IL (see next section), resulting in more ordered bilayers near the bilayer center. Interdigitation and the resulting higher order parameters also contribute to the increased thickness of the DSSM-containing bilayers. However, neither the thickness or the order profiles explain the higher concentration of CHL in the OL in the presence of DSSM, compared to DPSM.

CHL and SM form stable nanodomains in both the DPSM and DSSM systems, as apparent from 2D-density plots (Fig. 4C) and (Fig. 4D). Although phase separation is not complete, several nanodomains 2–3 nm in size are visible for both systems (Fig. 4). The pairing of the CHL headgroup and the SM headgroup is also apparent form radial distribution functions between the ROH bead of CHL and the sphingosine backbone bead AM1 of SM (Fig. 4A) and (Fig. 4B). The interactions of CHL with the AM1 bead of SM are comparable with the POPC glycerol backbone GL1 bead. The binding of CHL to PS lipids is weaker than the binding to either the POPC or SM head groups.

**Fig. 5:**
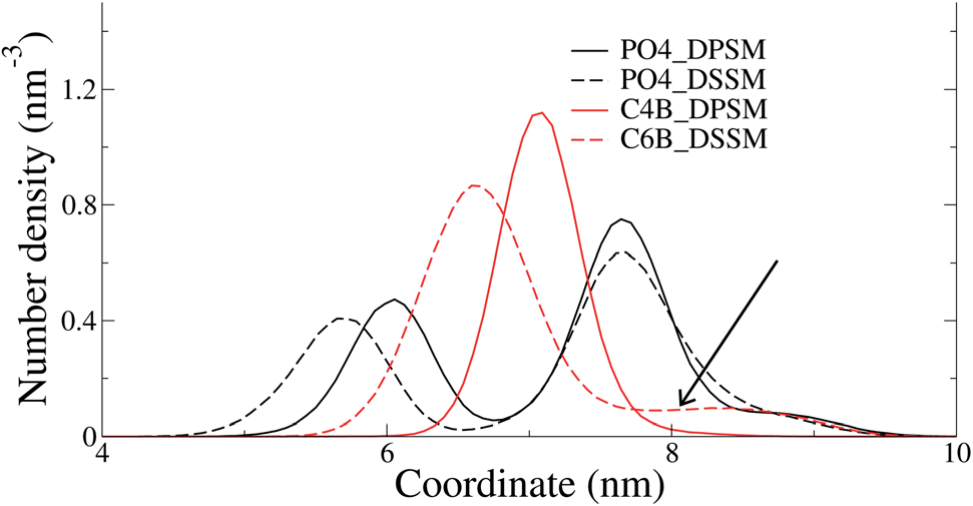
The density of the terminal SM bead along the bilayer normal for the DPSM and DSSM systems. The arrow highlights the tail in the distribution in the DSSM system resulting from the interdigitation of this bead into the opposing IL.

The C24 DSSM acyl chain protrudes into the opposing IL, while the C18 DPSM does not (Fig. 5). Since headgroup interactions, domain formation, thickness and order parameters are identical in the DPSM and DSSM systems. Furthermore, PS lipids, which are known to attract CHL to the IL^13^ are present in equal amounts in both the DPSM and DSSM systems. Therefore, we conclude that the interdigitation of lipid tails in the IL makes the IL denser, and reduces the free volume available for CHL. CHL is the only molecule which can spontaneously flip–flop in the simulations on the μs timescale, and therefore CHL accumulates in the OL when the C24 SM tail interdigitates into the IL. The hypothesis is in conflict with our previous findings, which were based on free energy calculations from all-atom simulations.^16^ The lipid composition in the said systems did not contain PS or PE lipids and therefore dissimilar from the ones we have used in the current CG simulations. More CHL in the OL (54%) was also observed in CG simulations of a bilayer with composition mimicking a plasma membrane.^25^ There are several key differences in the said simulations from our current work. Firstly, we have focussed on the differences between the effects of long-chain and short-chain SMs, while the systems in^25^ more resemble a realistic plasma membrane containing a mixture of short-chain and long-chain SM. Secondly, our systems are less likely to suffer from an initial area mismatch owing to reasons discussed earlier. Finally, our systems are much simpler in composition, with all lipids with an abundance of less than 5% being ignored.

The protein content in a real PM is nearly 30%, and CHL affinity to peripheral of specific regions of TM proteins can influence the overall distribution of CHL. Although inclusion of proteins could not be performed in the current set of simulations, our scheme of carefully equilibrating the systems to keep the areas of each monolayer constant can be used in the future to analyse complex membrane systems also containing proteins.

## 4 Conclusions

Overall, our data indicate that long-chain SM in the OL attracts more CHL to the OL, than short-chain SM does. There are, however, many other compositional determinants which will ultimately determine the CHL distribution ratio in both experiments and simulations. Since lipid composition has such an important role to play, the distribution of CHL will be sensitive to the cell and organelle type, as has been proposed in the past. Our simulations indicate that short-chain SM, when present in cells in diseased conditions, result in less CHL accumulation in the OL. A different CHL distribution can in principle affect nanodomain formation in complex membrane environments, and also influence signaling processes associated with these domains.

## Conflicts of interest

There are no conflicts to declare.

## Supplementary Material
